# Identification of deregulation of apoptosis and cell cycle in neuroendocrine tumors of the lung via NanoString nCounter expression analysis

**DOI:** 10.18632/oncotarget.3992

**Published:** 2015-05-04

**Authors:** Robert Fred Henry Walter, Robert Werner, Saskia Ting, Claudia Vollbrecht, Dirk Theegarten, Daniel Christian Christoph, Kurt Werner Schmid, Jeremias Wohlschlaeger, Fabian Dominik Mairinger

**Affiliations:** ^1^ Ruhrlandklinik, West German Lung Center, University Hospital Essen, University of Duisburg-Essen, Essen, Germany; ^2^ Institute of Pathology, University Hospital Essen, University of Duisburg-Essen, Essen, Germany; ^3^ Institute of Pathology, University Hospital Cologne, Cologne, Germany; ^4^ Department of Medical Oncology, West German Cancer Center, University Hospital Essen, University of Duisburg-Essen, Essen, Germany

**Keywords:** small-cell lung cancer, large-cell neuroendocrine lung cancer, carcinoids, apoptosis, NanoString nCounter

## Abstract

**Background:**

Neuroendocrine tumors of the lung comprise typical (TC) and atypical carcinoids (AC), large-cell neuroendocrine cancer (LCNEC) and small-cell lung cancer (SCLC). Cell cycle and apoptosis are key pathways of multicellular homeostasis and deregulation of these pathways is associated with cancerogenesis.

**Materials and Methods:**

Sixty representative FFPE-specimens (16 TC, 13 AC, 16 LCNEC and 15 SCLC) were used for mRNA expression analysis using the NanoString technique. Eight genes related to apoptosis and ten genes regulating key points of cell cycle were investigated.

**Results:**

*ASCL1, BCL2, CASP8, CCNE1, CDK1, CDK2, CDKN1A* and *CDKN2A* showed lower expression in carcinoids compared to carcinomas. In contrast, *CCNE1* and *CDK6* showed elevated expression in carcinoids compared to carcinomas. The calculated *BCL2/BAX* ratio showed increasing values from TC to SCLC.

Between SCLC and LCNEC *CDK2, CDKN1B, CDKN2A* and *PNN* expression was significantly different with higher expression in SCLC.

**Conclusion:**

Carcinoids have increased *CDK4/6* and *CCND1* expression controlling RB1 phosphorylation via this signaling cascade. *CDK2* and *CCNE1* were increased in carcinomas showing that these use the opposite way to control RB1. *BAX* and *BCL2* are antagonists in regulating apoptosis. *BCL2* expression increased over *BAX* expression with increasing malignancy of the tumor from TC to SCLC.

## INTRODUCTION

Neuroendocrine tumors (NET) of the lung comprise four entities with varying aggressiveness, ranging from well-differentiated typical carcinoids (TC), intermediate atypical carcinoids (AC) to poorly differentiated large-cell neuroendocrine carcinomas (LCNEC) and small-cell lung cancer (SCLC) [1–3]. The 5-year survival of the NET subgroups differ significantly with TC having the best survival rates (>87%) [4, 5], followed by AC with 60% [6] and LCNEC with 15-57% [7] and SCLC with less than 5% [8].

Cell cycle control and subsequent transcriptional regulation depends primarily on cyclin-dependent kinases (CDK), cyclin-dependent kinase inhibitors and cyclins that stimulate and inhibit each other in a highly timed manner to coordinate cell division and duplication [9]. The downstream target of these cell cycle mediators is RB1 (retinoblastoma 1, tumor suppressor) and chemotherapy efficacies were reported to depend on RB1 status in several cancer types [10–13]. New and specific cyclin inhibitors control the G1/S-phase checkpoint in RB1 active cells leading to a strong cytostatic effect in tumorous and non-tumorous cells [13, 14]. One of these substances is PD-0332991, which is tested as single agent and as combination therapy regimen in clinical settings [13, 14]. This small molecule inhibitor, a pyridopyrimidine, is highly selective for CDK4/6 [14].

Apoptosis is important for multicellular homeostasis and evasion of apoptosis is another hallmark of cancer [1, 15, 16]. Pro-apoptotic regulators as BAX, P53 and caspases drive apoptosis in contrast to anti-apoptotic regulators such as BCL2 [15, 17, 18].

Based on recent literature, this study was conducted to investigate the mRNA expression of apoptosis- and cell cycle-related genes and their impact on aggressiveness of NET of the lung by using the NanoString nCounter technology. The method allows enzyme-free digital detection of mRNAs, fusion genes or copy number variations (CNV) in a high-throughput fashion [19, 20]. Each investigated nucleic acid is targeted by a capture and a reporter probe that contain approximately 50 nucleotides (nt) that are complementary to the region of interest (ROI) [19, 21]. Each capture probe carries a biotin-tag for immobilization and each reporter probe is labeled with a barcode of six fluorophores that is unique for each targeted sequence. The fluorescent barcodes are imaged and the software counts and decodes the barcodes. In this way, up to 800 different targets can be detected simultaneously in one analysis [19, 21]. With this method, short target regions of approximately 100 nt are reliably detectable in formalin-fixed, paraffin-embedded tissue (FFPE) and fragments of 100 nt were reported to be detectable with nearly 100% efficiency [19, 22–24]. Importantly, Reis et al. proofed that the NanoString technique is able to analyze mRNA from FFPE tissue with results similar to fresh-frozen tissue [23]. This is of high interest, because FFPE is an important source for routine diagnostic in modern pathology connecting clinical follow-up data to large patient populations [22].

## RESULTS

Sixty tumor specimens were investigated including 16 TC (27%) and 13 AC (22%), 16 LCNEC (27%) and 15 SCLC (25%) cases. Twenty-seven female (45%) and 25 male patients (42%) were investigated. For eight patients the gender remained inconclusive.

**Figure 1:**
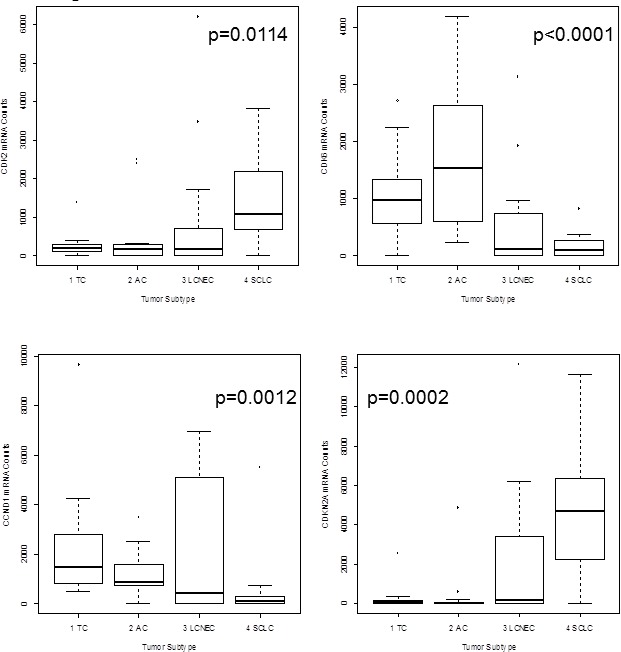
The boxplots show mRNA expression of *CDK2, CDK6, CCND1* and *CDKN2A* in neuroendocrine tumors of the lung On the x-axis the tumor subtype is shown and the y-axis indicates the amount of detected mRNA transcripts. *CDK2* expression was higher in high-grade NET with highest expression in SCLC, whereas *CDK6* showed significantly higher expression in carcinoids than in carcinomas. *CCND1* expression decreased from TC to SCLC. *CDKN2A* was identified as prominent marker for aggressiveness of the tumor. Only high-grade tumors and aggressive carcinoids showed elevated *CDKN2A* expression.

**Figure 2:**
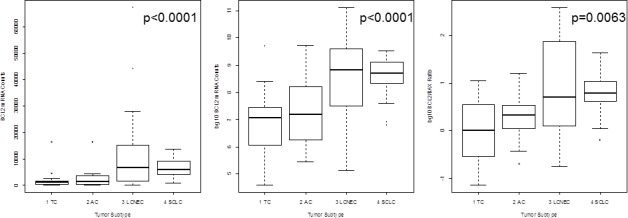
The mRNA expression of *BCL2* is shown in logarithmic and linear scale and additionally, the calculated *BCL2/BAX* ratio is charted in logarithmic scale The x-axis shows the tumor type, whereas the y-axis shows the detected mRNA transcripts. *BCL2* expression is minimal in TC and increased towards SCLC linking dedifferentiation with increasing proliferative activity. This is confirmed by the *BCL2/BAX* ratio showing that most TC have an equilibrium between *BCL2* (driver of proliferation) and *BAX* (driver of apoptosis), but increasing malignancy is accompanied by a reduction of apoptosis and increased proliferation.

*ASCL1* (p = 0.0016), *BCL2* (p < 0.0001), *CASP8* (p = 0.0030), *CCNE1* (p = 0.0135), *CDK1* (p = 0.0146), *CDK2* (p = 0.0114), *CDKN1A* (p = 0.0107) and *CDKN2A* (p = 0.0002) showed lower expression in carcinoids compared to carcinomas. In contrast, *CCND1* (p = 0.0012) and *CDK6* (p < 0.0001) showed elevated expression in carcinoids compared to carcinomas. The results for *CDK2, CDK6, CCND1* and *CDKN2A* are summarized in Figure 1. The calculated *BCL2/BAX* ratio (p = 0.0063) showed increasing values from TC to SCLC. The correlation of *BCL2* mRNA expression versus tumor type is shown in Figure 2 in logarithmic and linear scale. Additionally, the calculated *BCL2/BAX* ratio is shown in logarithmic scale in Figure 2.

Statistical analysis revealed a slightly higher expression of *CDKN1A* (p = 0.0485) and *PNN* (p = 0.0016) in AC compared to TC. Contrary, *CASP8* (p = 0.0393) was slightly increased in TC compared to AC.

**Figure 3:**
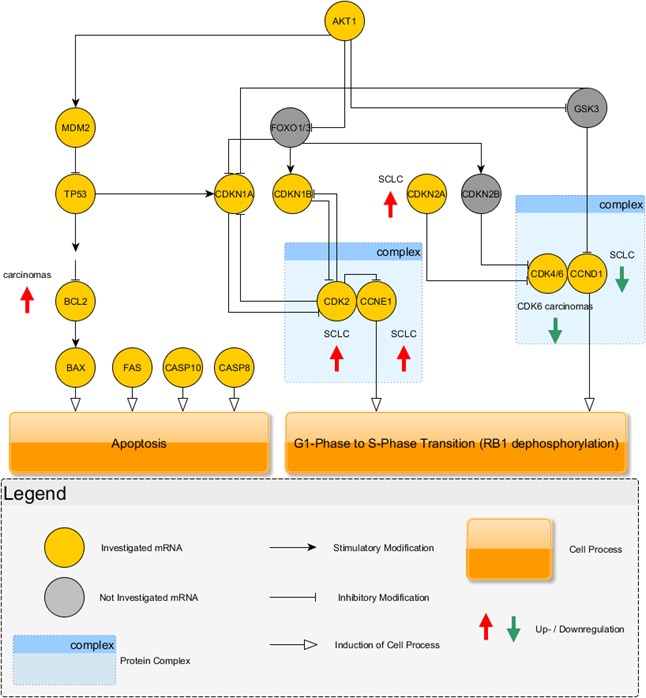
The investigated key mediators of apoptosis and cell cycle regulation are shown graphically Carcinoids use the *CDK4/6* complex in combination with CCND1 to drive cell cycle progression. Instead, carcinomas showed a downregulation of these cell cycle mediators and additionally, the inhibitor of this section, *CDKN2A*, was increased in carcinomas. Carcinomas showed higher expression of *CDK2* and *CCNE1* indicating that cell cycle progression is driven by this complex. Furthermore, carcinomas showed elevated expression of *BCL2*, an antagonist of apoptosis, which increased over *BAX* expression leading to insensitivity to apoptosis.

Between SCLC and LCNEC *CDK2* (p = 0.0142), *CDKN1B* (p = 0.0032), *CDKN2A* (p = 0.0291) and *PNN* (p = 0.0008) expression was significantly different with higher expression in SCLC. An overview of the results is charted in Figure 3.

Minimal *CCND1* (p = 0.0035) expression correlated with lymph node invasion whereas, for *CDKN2A* (p = 0.0002) minimal to absent expression correlated with N0-status. Additionally, downregulation of *CCND1* (p = 0.0234) expression to a minimum correlated with tumor-invasion into veins. For *BCL2* (p = 0.0391) lowered expression was found in V0-tumors.

## DISCUSSON

The NanoString technique was able to specifically and sensitively analyze 91 mRNA -target transcripts from all investigated FFPE samples by using 100 ng of total RNA per sample. The method relies on probes-pairs that are complementary to 100 nt of the region of interest making it an ideal method for the analysis of degraded archival FFPE tissue [19, 22–24].

Here we present the differences between carcinoids and carcinomas regarding cell cycle control and apoptosis leading to differences in the aggressiveness of the clincopathological subtypes.

**Figure 4:**
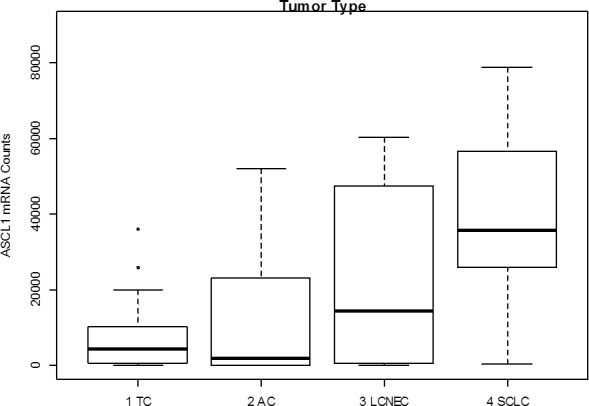
Gene expression levels of *ASCL1* in neuroendocrine tumors of the lung *ASCL1* shows very high counts over all investigated entities. Median mRNA counts increase with increasing malignancy having the highest median counts in SCLC.

Achaete-scute complex homolog 1 (Drosophila) (ASCL1) belongs to the basic helix loop helix transcription factor family and mediates neuroendocrine differentiation [25, 26]. Its expression is reported in fetal and adult neuroendocrine tissue and in various neuroendocrine tumors including SCLC [25, 26]. Furthermore, Miki et al. reported high expression in carcinoid tumors of the lung [27]. In the present study, *ASCL1* showed very high counts compared to the other investigated mRNA targets and was expressed in the majority of all NET of the lung with increasing expression from carcinoids to carcinomas (Figure 4). Until now, little is known about the progenitor cells of NET in the lung, but it is speculated that they may arise from pulmonary neuroendocrine cells (PNEC), which account for approximately 0.4% of bronchial epithelial cells, but drive prenatal development of the lung as well as development of chemoreceptors in the adult lung [2, 28–31]. Miki et al., suggested that ASCL1 may be a marker for early neuroendocrine differentiation in epithelial cells and that downregulation of *ASCL1* by RNA interference led to significant growth reduction in tumors with high ASCL1 expression [27]. In conclusion, ASCL1 seems to a driving mediator of neuroendocrine (de-)differentiation in several tissues and distinct developmental key points, but its diagnostical relevance is limited due to its widespread appearance in all entities of neuroendocrine tumors.

One hallmark of cancer is the evasion of apoptosis and P53 is one key mediator regulating BCL2 and BAX, but inactive P53 is unable to control apoptosis or proliferation via this mechanism [1]. P53 inactivation via genetic alteration has been widely studies in LCNEC and SCLC [2]. *TP53* mutations and loss of heterozygosity (LOH) of 17p13 are frequent events in LCNEC (>80%) and SCLC (>90%), but rare in carcinoids [2, 32]. BAX and BCL2 are antagonists regarding the regulation of apoptosis [1, 15]. BAX drives apoptosis whereas BCL2 is a suppressor of this cell function [1, 15]. A calculated ratio indicates the likelihood of cell to undergo apoptosis [33]. In the present paper, the median *BCL2/BAX* ratio for TC showed equilibrium between *BAX* and *BCL2* expression. With increasing malignancy, *BCL2* expression increased over *BAX* leading to lowered apoptosis in most high-grade tumors. Furthermore, *BCL2* showed significantly differential expression between carcinoids and carcinomas. Elevated expression was more common in carcinomas. In line with that, elevated expression of *BCL2* was a marker for invasiveness into veins.

Another hallmark of cancer is the deregulation of the cell cycle found in virtually all neoplasms [1, 16, 34–36]. Cell cycle progression and proliferation are mainly controlled by the tumor suppressor RB1 at the G1/S-phase checkpoint [34, 35, 37]. RB1 is controlled by two upstream regulatory signaling cascades of CDKs and cyclins [34, 35]. CDKN2A, CDK4/6 and CCND1 are direct upstream regulators on the one side of the signaling cascade and on the other side CDKN1B, CDKN1A, CDK2 and CCNE1 are controlling RB1 phosphorylation [34, 35, 37].

Based on our results, carcinoids seem to use the signaling cascade via upregulation of *CDK4/6* and *CCND1* expression to control RB1 phosphorylation. Additionally, *CDKN2A*, the regulatory inhibitor of *CDK4/6* [35], shows minimal to no expression in carcinoids. Palbociclib (PD-0332991), a pyridopyrimidine, is a highly selective CDK4/6 inhibitor that stops cell cycle progression at the G1/S-phase checkpoint in several *in vivo* and *in vitro* models and is currently tested in clinical settings [13, 14], indicating a potential, new therapy approach for carcinoid tumors of the lung. Until now, somatostatin analogues, such as octreotide acetate, were identified as effective compounds for patients with carcinoid tumors [38] and may show an enhanced effect in combination with cyclin inhibitors, because a cumulative effect was reported when therapy regimen contained palbociclib in combination with other drugs [13]. Flavopiridol, is a semisynthetic alkaloid and potent CDK inhibitor also inhibiting CDK6 and showed stronger induction of apoptosis and reduction of cell viability when combined with another cytostatic drug in an *in vivo* model [39].

For NSCLC a downregulation of *CDKN2A* was reported [36] prooving that carcinoids use a similar way as NSCLC to drive G1/S-phase transition, instead cell cycle progression in LCNEC and SCLC is driven by the contrary pathway as summarized in Figure 3. The investigated carcinomas used the signaling cascade via increased expression of *CDK2* and *CCNE1* to drive cell cycle transition. High-grade tumors, especially SCLC, are associated with smoking [16] leading to an inactivation of P53 in up to 100% of all high-grade tumors [1]. CDKN1A is regulated by P53, but as reported, P53 activity is missing in nearly all high-grade tumors showing that this contributes to the use of the CDK2-CCNE1-pathway, because a downregulation of this pathway via CDKN1A is lacking. Flavopiridol also inhibits CDK2 [37] and may inhibit cell cycle progression in high-grade NETs.

*CDKN2A* showed nearly no expression in low-grade tumors but highly elevated expression was found in most LCNEC and SCLC. Interestingly, one of the investigated AC showed a high expression of approximately 5,000 *CDKN2A* mRNA counts and showed similar clinical behavior as an SCLC with fast progression of the disease and early death. Therefore, elevated *CDKN2A* expression seems to be a potent biomarker for aggressiveness of neuroendocrine tumors of the lung, regardless of the subgroup. Additionally, expression of more than approximately 480 mRNA counts was found as threshold for a potential lymph node invasion. Similarly, high expression of CDKN2A on the protein level correlated to poor differentiation, high grade and poor prognosis in advanced ovarian carcinomas and gastrointestinal stromal tumors [40, 41]. For other malignancies, downregulation of CDKN2A expression was reported to correlate with poor outcome and high TNM stage [42], indicating a tissue and tumor specific regulation.

In contrast, *CCND1* showed lowered expression in most LCNEC and in nearly all SCLC showing an inverted correlation to the malignancy of the tumors. Furthermore, minimal to absent expression was a marker for aggressiveness of the tumors regarding lymph node and vein invasion. In other malignancies, RB1 inactivation correlates with overexpression of *CCND1* and downregulation of *CDKN2A* [43].

In summary, deregulation of apoptosis and cell cycle mediators is an early event in several human cancers containing promising potential biomarkers amongst them with important impact on clinical decisions, such as prognosis, treatment and potential new therapy targets [44–47].

## MATERIALS AND METHODS

Representative specimens of each tumor entity (16 TC and 13 AC, 16 LCNEC and 15 SCLC) were used for mRNA expression analysis. Patients that received chemotherapy before resection of tumor tissue specimens were excluded. The initial diagnosis was reevaluated by two experienced pathologists (JWO, DTH). Additional inclusion criteria were sufficient tumor material (at least 80% tumor cells) and minimal contamination by benign and stromal cells (< 20% stromal cells/lymphocytes). Specimens were collected at the tumor bank at the Institute of Pathology, University Hospital Essen (Germany) from 2005 till 2012. Tumor classification is based on the *WHO Classification Of Tumours* guidelines (2004) [48] and TNM-staging is based on the *UICC Classification of Malignant Tumours* [49]. Clinical data were obtained from the patients' records. The study was conducted retrospectively for the identification of biomarkers to identify deregulation in neuroendocrine tumors regarding apoptosis and cell cycle. The study was approved by the ethical committee of the University Hospital Essen (ID: 13-5382-BO). The investigation conforms to the principles outlined in the declaration of Helsinki.

### RNA extraction and RNA integrity assessment

Three to five paraffin sections with a thickness of 4 μm per sample were deparaffinized with xylene prior to RNA extraction using the RNeasy FFPE kit (Qiagen, Hilden, Germany) according to the manufacturer's recommendations. Total RNA concentrations were measured using a Nanodrop 1000 instrument (Thermo Fisher Scientific, Waltham, USA). RNA integrity was assessed using an Agilent 2100 Bioanalyzer (Agilent Technologies, Santa Clara, USA) at the NanoString nCounter Core Facility at the University of Heidelberg, Germany. Smear analysis was performed using the Agilent 2100 expert software to determine the proportion of RNA ≥300 nt within a given sample.

### NanoString codeSet design and expression quantification

Various relevant genes involved in different tumor-associated signaling pathways and neuroendocrine differentiation were included in a custom CodeSet. The CodeSet was designed to contain a total of 91 genes with different signature genes for each subgroup (Table 1). Nine genes associated with apoptosis (*ASCL1, BAX, BCL2, CASP8, CASP10, FAS, MDM2, P53, PNN*) and ten genes involved in cell cycle regulation (*CCND1, CCNE1, CDK1, CDK2, CDK4, CDK6, CDKN1A, CDKN1B, CDKN2A, MIB1*) were investigated. Three potential reference genes (*ACTB, GAPDH, and HPRT1*) were also included in the CodeSet for biological normalization. Probe sets for each gene in the CodeSet were designed and synthesized at NanoString Technologies, Seattle, USA.

**Table 1:** A total of 91 mRNA targets including 12 cell processes were investigated using a NanoString nCounter custom CodeSet

Cell Process	Investigated Targets
Sox signaling	*MYB, MYBBP1A, OCT4, PAX6, PCDHB, RBP1, SDCBP, SOX2, SOX4, SOX11, TEAD2*
MET pathway	*GAB1, GRB2, MET, MST1R, PAX5*
mTOR signaling	*MTOR, RHOA, RICTOR, RPTOR*
angiogenesis	*CRHR2, FIGF, FLT4, HIF1A, KDR, MMP3*
apoptosis	*ASCL1, BAX, BCL2, CASP8, CASP10, FAS, MDM2, TP53, PNN*
neuroendocrine differentiation	*CHGA, GABBR2, NCAM1, NTS, RTN1, SEMA3B, SYP*
folate metabolism	*ATIC, DHFR, FOLR1, FPGS, GART, GGT1, SLC19A1, TYMS*
DNA-repair	*ERCC1, MLH1, MSH2, MSH6, XRCC1*
cell cycle regulation	*CCND1, CCNE1, CDK1, CDK2, CDK4, CDK6, CDKN1A, CDKN1B, CDKN2A, MIB1*
tumor environment	*LDHA, LDHB, LDHC, MAN2A1, MAN2B1, MAN2C1, TKTL1*
growth factors and signaling	*IGF1, IGF2, EGFR, FGFR1, AKT1, ALK, PTEN*
additional genes	*CAT, CYP1A1, FN1, NES, NKX21, SOD1, STK11, TWSG1, UCHL1*
Rference Genes	*ACTB, GAPDH, HPRT1*

Total RNA (100 ng) including miRNA from FFPE material was analyzed at the NanoString nCounter Core Facility at the University of Heidelberg, Germany.

### NanoString data processing and statistical analysis

Raw NanoString counts for each gene were subjected to a technical normalization considering the counts obtained for positive control probe sets. After the technical normalization, a biological normalization using the three reference genes included in the CodeSet was performed.

All statistical analyses were performed with the R statistical programming environment (v2.15.2). For dichotomous factors such as gender and expression level the Wilcoxon Mann-Whitney rank sum test was applied. The Kruskal-Wallis test was used to correlate tumor type and gene expression. The same test was also used to perform subgroup analysis between low- and high-grade tumors, atypical and typical carcinoids and SCLC versus LCNEC. Correlations between gene expression and TNM-stages were analyzed by Spearman's rank correlation test.

The level of statistical significance was defined as p≤0.05.
